# Effects of illness perceptions on health-related quality of life in patients with rheumatoid arthritis in China

**DOI:** 10.1186/s12955-021-01770-4

**Published:** 2021-04-20

**Authors:** Juan Wang, Zhe Yang, Yan Zheng, Yaling Peng, Qing Wang, Hongli Xia, Yan Wang, Jin Ding, Ping Zhu, Lei Shang, Zhaohui Zheng

**Affiliations:** 1 Department of Clinical Immunology, Xijing Hospital, Fourth Military Medical University, Xi’an, 710032 Shaanxi Province China; 2 Department of Health Statistics, Fourth Military Medical University, Xi’an, 710032 Shaanxi Province China

**Keywords:** Rheumatoid arthritis, Illness perceptions, Health-related quality of life (HRQoL), SF-36

## Abstract

**Objectives:**

For patients with rheumatoid arthritis (RA) in China, little is known of how their illness perceptions affect their health-related quality of life (HRQoL). The present study investigated associations between specific illness perceptions due to RA and HRQoL features.

**Methods:**

For 191 patients with RA, illness perceptions were measured using the Brief Illness Perceptions Questionnaire (BIPQ) comprising 8 domains. HRQoL was determined with the Medical Outcomes Study 36-Item Short-Form Health Survey (SF-36). Multivariate linear regression analyses were performed.

**Results:**

The overall BIPQ of patients with RA was 49.09 ± 11.06. The highest and lowest scores were for concern (9.15 ± 1.81) and personal control (4.30 ± 2.52), respectively. Multivariate stepwise regression analyses showed that the overall BIPQ was significantly negatively associated with each HRQoL feature, and HRQoL total score (β = − 0.343, *P* < 0.001, 95% CI − 7.080 to − 4.077). Positive associations between BIPQ features and HRQoL included personal control (β = 0.119, *P* = 0.004, 95% CI 2.857–14.194) and treatment control (β = 0.084, *P* = 0.029, 95% CI 0.640–12.391). Negative associations with HRQoL were identity (β = − 0.105, *P* = 0.034, 95% CI − 13.159 to − 0.430) and emotional response (β = − 0.207, *P* < 0.001, 95% CI − 18.334 to − 6.811).

**Conclusions:**

Patients with RA in China perceive their illness in ways that affect their HRQoL. These results suggest that strategies that target these perceptions may improve the quality of life of these patients.

## Introduction

Rheumatoid arthritis (RA) is a chronic disease characterized by symmetric polyarticular arthritis. It is the most common autoimmune disease. Clinical manifestations of RA include joint swelling, pain, and limited function [1, 2]. Progression of RA may eventually lead to joint deformities which seriously affect patients’ quality of life [3, 4]. In RA, each patient's presentation and course of disease is unique. To understand the individualized course of chronic diseases, there has been growing interest in common sense models. These tested models suggest that the patient's perception of illness, that is, their own cognitive and emotional responses, direct their response to that illness [5–9]. Individuals actively try to make sense of their symptoms and form personal beliefs about their illness. These beliefs, in turn, determine their subsequent coping behavior and quality of life [10].

Health-related quality of life (HRQoL) may be measured to reveal the physiological, psychological, and social functions of patients with RA [3, 11, 12]. Illness perception is associated with quality of life, social function, and disease prognosis [13–18]. Such knowledge is applied to guide clinicians in the management of RA, to improve the HRQoL and the prognosis of their patients. In rheumatology, the effect of illness perception has been studied extensively in western populations, specifically for RA [19, 20], systemic sclerosis [21], lupus nephritis [22, 23], psoriatic arthritis [16], multiple sclerosis [24], and systemic lupus erythematosus [25, 26]. Research has highlighted the importance of the beliefs of patients with RA about their illness and symptoms as they affect their HRQoL. The identification of these patients’ perceptions could positively influence quality of life, as illness perception is amenable to intervention [27].

However, to our knowledge, there have been no studies on illness perception and its association with HRQoL for patients with RA in China. The purpose of the present study was to identify those perceptions of illness that influence patients’ quality of life, to better guide clinicians in the management of RA, and improve the ability of patients with RA to self-manage and improve their HRQoL.

## Methods

### Study design and data collection

This cross-sectional study was conducted in an outpatient clinic at Xijing Hospital, Xi’an, Shaanxi, China from March 2017 to December 2017. The Ethics Committee of Xijing Hospital approved the study (KY20140902-5), and all subjects provided written informed consent prior to their participation.

The study included patients with RA diagnosed according to the ACR (American College of Rheumatology)/EULAR (European League against Rheumatism) 2010 classification criteria [28]. All the patients were at least 18 years of age; able to understand and communicate in Chinese; and willing to participate. Patients with any of the following were excluded: suffering from other chronic diseases; recent major surgery; unstable condition; or intellectual or cognitive impairments. This study is based on the infinite population sampling formula:$n = \left( {{{u_{{{\alpha /2}}} \sigma } /\delta }} \right)^{2}$, where $u_{{{\alpha /2}}} = 1.96$, $\sigma = 5$, and $\delta = 0.7$. The values $\sigma$ and $\delta$ refer to the literature related to illness perceptions [27]; the sample size was calculated as 196.

Sociodemographic and clinical data were collected through face-to-face interviews with the patients. Disease activity estimates were based on the Disease Activity Score 28 (DAS28): an index of physician-rated tenderness and swelling scores for 28 joints and an inflammatory biomarker (erythrocyte sedimentation rate or C-reactive protein) CRP as the inflammatory index [29]. Illness perceptions and HRQoL were assessed via patient-reported outcome measures.

### Measurements of illness perceptions

The Brief Illness Perceptions Questionnaire (BIPQ) assesses an individual’s perceptions and cognitions regarding their disease [7, 30]. The Chinese BIPQ has been tested and validated previously [31, 32]. The questionnaire measures the following 8 domains of illness perception: consequences; timeline; personal control; treatment control; identity; concern; coherence; and emotional response. The 8 BIPQ domains that together reflect an individual’s perception of their disease. The score of each domain may range from 0 to 10. The overall BIPQ score ranges from 0 to 80, a higher score reflects a more negative view of the illness.

### Measurements of HRQoL

HRQoL was measured with the Chinese version of the Medical Outcomes Study 36-item Short-Form Health Survey (SF-36). The SF-36 consists of 36 items that measure the following 8 dimensions: physical function (PF); role limitations related to physical problems (RP); bodily pain (BP); general health perception (GH); vitality (VT); social functioning (SF); role limitations due to emotional problems (RE), and mental health (MH). The score of each dimension is converted to a standard score ranging from 0 to 100, with a highest score indicating the best HRQoL [33]. The SF-36 has shown good reliability and validity among various Chinese patient populations [34, 35].

### Statistical analysis

Statistical analyses were performed using Statistical Package for Social Science 18.0 (SPSS, Chicago, IL) software. The descriptive statistics are presented as the mean and standard deviation for quantitative data, and percentage for count data. The independent samples *t*-test and one-way analysis of variance were used to analyze inter-group differences with normal distribution.

Linear regression analyses were used to test the univariate correlations between the domains of illness perception and HRQoL, and to screen the significant independent variables (*P* < 0.1) for subsequent multivariate regression analyses. Multivariate stepwise regression analysis was used to explore the effect of illness perceptions on HRQoL. After controlling for demographics and disease characteristics, independent variables were entered stepwise into the model. *P* values < 0.1 were added into the regression, and only *P* values < 0.05 were considered statistically significant. The total score for quality of life was normally distributed (*P* = 0.066); the scores of each dimension did not conform to a normal distribution and were analyzed after normalization conversion (*P* < 0.001).

## Results

### Patient characteristics

The questionnaires were distributed to 200 eligible patients. Because 9 questionnaires were missing data, there were finally 191 study participants. The average age of the participants was 45.06 ± 13.32 years, 140 (73.30%) were women (Table 1), 37.7% had a disease duration of more than 5 years, 26.18% were in remission, and 16.75% had severe disease.

**Table 1:** Baseline characteristics of the patients (n = 191)^a^

Gender	Male	51 (26.70)
	Female	140 (73.30)
Age, y		45.06 ± 3.32
Ethnic group	Han	182 (95.29)
	Hui	8 (4.19)
	Other	1 (0.52)
Marital status	Married	168 (87.96)
	Other	23 (12.04)
Education	≤ Junior middle school (9 y)	82 (42.93)
	Senior middle school (12 y)	41 (21.47)
	≥ College (15 y)	68 (35.60)
Residence	Rural	95 (49.74)
	Urban	96 (50.26)
Income/month^b^	< 1000 yuan	45 (23.56)
	1000–3000 yuan	94 (49.21)
	> 3000 yuan	52 (27.23)
Occupation	Unemployed	76 (39.79)
	Blue collar	39 (20.42)
	White collar	76 (39.79)
Disease duration, y		5.73 ± 7.33
DAS28		3.58 ± 1.32

^a^Reported as n (%) unless indicated otherwise

^b^Family income/monthly

### Illness perceptions

The overall BIPQ score of the participants was 49.09 ± 11.06. The scores of each dimension of the BIPQ were as follows: concern (9.15 ± 1.81); timeline (7.68 ± 2.78); treatment control (7.83 ± 2.3); emotions (7.15 ± 2.9); consequences (6.72 ± 3.02); identity (6.61 ± 2.74); coherence (6.08 ± 2.60); and personal control (4.30 ± 2.52). Concern and timeline received the highest, while personal control was the lowest.

### HRQoL

For the unstratified (overall) population, the SF-36 scores of the 8 dimensions, from highest to lowest, were social functioning, mental health, physical function, vitality, bodily pain, general health perception, role limitations due to emotional problems, and role limitations related to physical problems. Compared with the younger subgroup (age < 45 y), the older subgroup had significantly lower scores for physical function, vitality, and social functioning (*P* < 0.05, = 0.018, and = 0.039, respectively). When stratified by occupation (unemployed, blue, and white collar) and 3 levels of education, each dimension differed significantly among the subgroups except for role limitations due to emotional problems. Significant differences in SF-36 scores based on disease duration were found in all dimensions except general health perception, role limitations due to emotional problems, and mental health. When the population was stratified by DAS28 (remission, low, moderate, or high), each dimension differed significantly among these subgroups (*P* < 0.05, Table 2).

**Table 2:** SF-36 scores for 8 dimensions reflecting HRQoL^a^ by various population stratifications (n = 191)

	PF	RP	BP	GH	VT	SF	RE	MH
*Gender*								
Male	54.22 ± 33.46	19.61 ± 35.47	41.96 ± 26.79	34.08 ± 20.72	45.88 ± 24.08	70.34 ± 32.30	25.49 ± 40.87	62.12 ± 21.25
Female	60.11 ± 27.80	23.21 ± 37.17	47.39 ± 26.16	37.67 ± 20.32	52.75 ± 22.71	76.61 ± 31.91	33.10 ± 40.87	60.40 ± 23.41
*P*	0.264	0.549	0.209	0.284	0.070	0.233	0.257	0.646
*Age, y*								
< 45	69.04 ± 24.53 ^b^	25.28 ± 36.05	49.29 ± 23.14	39.47 ± 18.64	55.17 ± 21.25 ^b^	80.06 ± 28.75 ^b^	34.08 ± 40.19	61.84 ± 21.86
≥ 45	49.36 ± 30.34	19.61 ± 37.17	43.01 ± 28.69	34.30 ± 21.68	47.21 ± 24.32	70.47 ± 34.19	28.43 ± 41.53	60.00 ± 23.69
*P*	< 0.001	0.287	0.096	0.078	0.018	0.039	0.342	0.579
*Education*								
≤ Junior middle school	46.71 ± 29.88 ^b^	14.63 ± 33.09 ^b^	36.32 ± 25.81 ^b^	31.71 ± 22.34 ^b^	42.20 ± 25.03 ^b^	64.33 ± 34.31 ^b^	23.17 ± 37.29	52.59 ± 24.81 ^b^
Senior middle school	62.32 ± 29.63	26.22 ± 39.51	47.59 ± 26.64	40.37 ± 20.89	56.95 ± 19.97	80.18 ± 29.04	36.59 ± 46.43	68.10 ± 21.46
≥ College	70.51 ± 23.01	29.04 ± 37.81	56.54 ± 22.67	40.54 ± 16.33	57.79 ± 19.25	84.56 ± 27.16	37.25 ± 40.53	66.47 ± 17.58
*P*	< 0.001	0.041	< 0.001	0.013	< 0.001	< 0.001	0.068	< 0.001
*Residence*								
Rural	53.84 ± 30.20	20.79 ± 36.04	39.97 ± 25.61 ^b^	33.25 ± 21.54 ^b^	47.58 ± 25.33	71.57 ± 35.84	31.23 ± 42.33	57.09 ± 25.11 ^b^
Urban	63.18 ± 28.06	23.70 ± 37.41	51.84 ± 25.90	40.14 ± 18.77	54.22 ± 20.54	78.26 ± 27.58	30.90 ± 39.66	64.58 ± 19.72
*P*	0.002	0.020	0.048	0.151	0.956	0.023	0.134	0.585
*Income/month, yuan*^c^								
< 1000	47.89 ± 32.66 ^b^	13.33 ± 32.25	38.42 ± 26.51 ^b^	31.13 ± 24.78	41.67 ± 25.02 ^b^	64.72 ± 34.78 ^b^	23.70 ± 40.59	53.96 ± 26.09 ^b^
1000–3000	59.57 ± 27.50	23.40 ± 37.35	44.35 ± 25.27	36.99 ± 19.09	52.39 ± 22.51	76.33 ± 32.56	30.85 ± 40.08	61.91 ± 21.99
> 3000	65.87 ± 27.81	27.88 ± 38.24	55.31 ± 25.99	41.04 ± 17.73	56.25 ± 20.88	81.25 ± 26.72	37.82 ± 42.28	64.92 ± 20.24
*P*	0.009	0.137	0.005	0.057	0.005	0.033	0.238	0.050
*Occupation*								
Unemployed	48.36 ± 28.69 ^b^	13.49 ± 32.26 ^b^	37.93 ± 27.00 ^b^	32.29 ± 20.26 ^b^	43.82 ± 24.00 ^b^	67.11 ± 34.45 ^b^	23.68 ± 38.80	55.21 ± 25.91 ^b^
Blue collar	59.49 ± 29.49	19.87 ± 34.02	39.67 ± 24.63	35.13 ± 24.12	49.10 ± 23.48	70.19 ± 32.77	32.48 ± 42.22	57.54 ± 22.06
White collar	68.22 ± 27.05	32.24 ± 39.96	57.16 ± 22.64	41.95 ± 17.43	58.95 ± 19.82	85.20 ± 26.28	37.72 ± 41.58	68.21 ± 17.55
*P*	< 0.001	0.006	< 0.001	0.012	< 0.001	0.001	0.103	0.001
*Disease duration, y*								
≤ 1	61.60 ± 29.62 ^b^	23.96 ± 39.01 ^b^	48.36 ± 28.16 ^b^	39.79 ± 21.43	54.17 ± 24.97 ^b^	80.73 ± 33.76 ^b^	36.57 ± 44.11	62.17 ± 24.60
1–5	66.06 ± 26.74	35.11 ± 42.56	55.83 ± 23.92	38.40 ± 17.46	57.45 ± 20.32	84.57 ± 28.10	36.17 ± 44.95	64.00 ± 20.24
> 5	50.56 ± 29.45	12.15 ± 26.20	37.06 ± 23.38	32.53 ± 20.76	43.40 ± 21.36	62.85 ± 29.22	22.22 ± 33.10	57.50 ± 22.41
*P*	0.010	0.003	< 0.001	0.083	0.002	< 0.001	0.066	0.262
*DAS28*^d^								
Remission	82.70 ± 14.82 ^b^	58.50 ± 44.50 ^b^	75.52 ± 13.33 ^b^	48.92 ± 16.06 ^b^	65.40 ± 18.59 ^b^	97.75 ± 24.57 ^b^	62.00 ± 40.41 ^b^	71.28 ± 17.50 ^b^
Low	72.16 ± 18.01	20.10 ± 30.01	53.47 ± 16.93	44.37 ± 19.12	61.27 ± 17.60	90.93 ± 19.86	35.29 ± 43.93	69.10 ± 17.78
Moderate	49.66 ± 20.75	4.31 ± 15.63	30.57 ± 16.53	29.28 ± 17.84	42.16 ± 18.94	60.34 ± 25.02	10.92 ± 24.49	52.48 ± 22.00
High	15.16 ± 17.71	1.56 ± 6.15	15.56 ± 10.81	18.91 ± 14.47	27.66 ± 19.55	40.23 ± 25.55	12.50 ± 27.76	46.63 ± 26.05
*P*	< 0.001	< 0.001	< 0.001	< 0.001	< 0.001	< 0.001	< 0.001	< 0.001
Overall	58.53 ± 29.44	22.25 ± 36.67	45.94 ± 26.37	36.71 ± 20.44	50.92 ± 23.22	74.93 ± 32.05	31.06 ± 40.91	60.86 ± 22.81

^a^Abbreviations for the 8 dimensions of the SF-36: *BP* bodily pain, *GH* general health, *MH* mental health, *PF* physical function, *RE* role limitations due to emotional problems, *RP* role limitations related to physical problems, *SF* social functioning

^b^*P* < 0.05

^c^Family income/monthly

^d^DAS28: clinical remission, < 2.6; low, 2.6–3.2; moderate 3.2–5.1; high, > 5.1

### Association between illness perception and HRQoL

Based on the linear regression analysis of the SF-36 dimensions, age and disease duration were each significantly associated with every SF-36 dimension, except mental health; and DAS28 was significantly associated with every SF-36 dimension (Table 3).

**Table 3:** Linear regression analysis of SF-36 dimensions (n = 191)

	PF	RP	BP	GH	VT	SF	RE	MH	Total SF-36
*Age*									
*R*^*2*^	0.166	0.025	0.045	0.037	0.068	0.053	0.035	0.007	0.078
*β*	− 0.047	− 0.159	− 0.211	− 0.193	− 0.261	− 0.229	− 0.187	− 0.085	− 0.280
***P***	**0.001**	**0.028**	**0.003**	**0.007**	**0.001**	**0.001**	**0.009**	**0.244**	**0.001**
*Disease duration*									
*R*^*2*^	0.089	0.033	0.058	0.048	0.061	0.063	0.028	0.000	0.069
*β*	− 0.298	− 0.182	− 0.241	− 0.218	− 0.247	− 0.251	− 0.168	− 0.016	− 0.263
***P***	**0.001**	**0.012**	**0.001**	**0.002**	**0.001**	**0.001**	**0.020**	**0.829**	**0.001**
*DAS28*									
*R*^*2*^	0.619	0.270	0.672	0.317	0.412	0.512	0.210	0.232	0.632
*β*	− 0.787	− 0.520	− 0.820	− 0.563	− 0.641	− 0.716	− 0.459	− 0.481	− 0.795
***P***	**0.001**	**0.001**	**0.001**	**0.001**	**0.001**	**0.001**	**0.001**	**0.001**	**0.001**
*BIPQ–1 Consequences*									
*R*^*2*^	0.221	0.102	0.249	0.187	0.231	0.199	0.073	0.142	0.266
*β*	− 0.470	− 0.319	− 0.499	− 0.433	− 0.481	− 0.446	− 0.270	− 0.377	− 0.516
***P***	**0.001**	**0.001**	**0.001**	**0.001**	**0.001**	**0.001**	**0.001**	**0.001**	**0.001**
*BIPQ–2 Timeline*									
*R*^*2*^	0.055	0.083	0.111	0.100	0.057	0.085	0.066	0.013	0.114
*β*	− 0.235	− 0.288	− 0.334	− 0.317	− 0.240	− 0.291	− 0.256	− 0.114	− 0.338
***P***	**0.001**	**0.001**	**0.001**	**0.001**	**0.001**	**0.001**	**0.001**	**0.115**	**0.001**
*BIPQ–3 Personal control*									
*R*^*2*^	0.113	0.031	0.090	0.142	0.102	0.104	0.059	0.038	0.127
*β*	0.336	0.175	0.299	0.377	0.320	0.322	0.243	0.196	0.356
***P***	**0.001**	**0.015**	**0.001**	**0.001**	**0.001**	**0.001**	**0.001**	**0.007**	**0.001**
*BIPQ–4 Treatment control*									
*R*^*2*^	0.006	0.014	0.002	0.023	0.010	0.007	0.025	0.004	0.018
*β*	0.076	0.120	0.049	0.153	0.101	0.085	0.159	0.059	0.134
***P***	**0.298**	**0.097**	**0.499**	**0.035**	**0.165**	**0.242**	**0.028**	**0.414**	**0.066**
*BIPQ–5 Identity*									
*R*^*2*^	0.257	0.174	0.300	0.173	0.221	0.244	0.138	0.126	0.330
*β*	− 0.507	− 0.417	− 0.547	− 0.416	− 0.470	− 0.494	− 0.372	− 0.355	− 0.574
***P***	**0.001**	**0.001**	**0.001**	**0.001**	**0.001**	**0.001**	**0.001**	**0.001**	**0.001**
*BIPQ–6 Illness concern*									
*R*^*2*^	0.008	0.020	0.023	0.028	0.021	0.007	0.017	0.006	0.025
*β*	− 0.090	− 0.141	− 0.151	− 0.166	− 0.146	− 0.082	− 0.131	− 0.079	− 0.158
***P***	**0.216**	**0.052**	**0.037**	**0.022**	**0.044**	**0.261**	**0.071**	**0.276**	**0.029**
*BIPQ–7 Coherence*									
*R*^*2*^	0.030	0.000	0.008	0.000	0.002	0.016	0.001	0.019	0.009
*β*	0.174	− 0.018	0.090	− 0.003	0.048	0.128	0.038	0.137	0.093
***P***	**0.016**	**0.802**	**0.216**	**0.963**	**0.511**	**0.078**	**0.601**	**0.059**	**0.203**
*BIPQ–8 Emotional response*									
*R*^*2*^	0.235	0.158	0.276	0.245	0.270	0.282	0.165	0.266	0.376
*β*	− 0.484	− 0.397	− 0.525	− 0.495	− 0.519	− 0.531	− 0.406	− 0.515	− 0.613
***P***	**0.001**	**0.001**	**0.001**	**0.001**	**0.001**	**0.001**	**0.001**	**0.001**	**0.001**
*Overall BIPQ*									
*R*^*2*^	0.350	0.206	0.386	0.334	0.333	0.354	0.210	0.212	0.476
*β*	− 0.592	− 0.454	− 0.621	− 0.578	− 0.577	− 0.595	− 0.458	− 0.461	− 0.690
***P***	**0.001**	**0.001**	**0.001**	**0.001**	**0.001**	**0.001**	**0.001**	**0.001**	**0.001**

We use only variables with 0.1 from above and we selection of independent variables for subsequent multiple regression analyses was based on these analyses

The overall BIPQ score was significantly associated with every SF-36 dimension, as was the following individual BIPQ domains: consequences, personal control, identity, and emotional response. Timeline was significantly associated with every SF-36 dimension, except mental health. Treatment control was associated with general health perception and role limitations due to emotional problems. Illness concern was associated with bodily pain, general health perception, and vitality. Coherence was associated only with physical function. Only the BIPQ domains treatment control and coherence were not significantly associated with the total SF-36 score (Table 3).

To evaluate the effects of certain demographics and illness perceptions on various dimensions of HRQoL in Chinese patients with RA, each HRQoL dimension was taken as a dependent variable, while the general characteristics and all BIPQ dimensions were considered independent variables in the multivariate stepwise regression analysis of HRQoL. According to the results (Tables 2 and 3), only the variables with a significant association (*P* < 0.1) were selected as the independent variables for multivariate stepwise regression (Table 4).

**Table 4:** Multiple linear regression analysis of 4 models^a^ of demographic and illness perceptions by SF-36 HRQoL dimensions^b^

	PF	RP	BP	GH	VT	SF	RE	MH	Total SF-36
*Model 1*									
*R*^*2*^	0.203	0.049	0.142	0.068	0.156	0.101	0. 035	0.108	0.171
Age	− 0.369; < 0.001; [− 1.104 to − 0.525]	–	− 0.149; 0.032; [− 0.565 to − 0.031]	− 0.158; 0.029; [− 0.461 to − 0.030]	− 0.225; 0.001; [− 0.597 to − 0.125]	− 0.185; 0.009; [− 0.781 to − 0.111]	− 0. 187; 0.009; [− 1.001 to − 0.143]	–	− 0.237; 0.001; [− 5.016 to − 1.393]
Education ^c^									
Senior middle school	–	–	–	–	0.183; 0.007; [3.483 to 18.494]	–	–	0.198; 0.005; [3.414 to 18.571]	0.159; 0.019; [11.541 to 127.202]
College	–	–	–	–	–	–	–	–	–
Occupation ^d^									
Blue collar	–	–	–	–	–	–	–	–	–
White collar	0.196;0.004; [3.87 to 19.599]	0.222; 0.002; [6.132 to 27.037]	0.318; < 0.001; [8.738 to 23.374]	0.178; 0.014; [0.653 to 12.464]	0.258; < 0.001; [6.313 to 19.104]	0.225; 0.002; [5.578 to 23.749]	–	0.285; < 0.001; [6.890 to 19.604]	0.281; < 0.001; [53.764 to 152.4]
*Model 2*									
*R*^*2*^	0.646	0.270	0.700	0.317	0.437	0.524	0.210	0.284	0.650
Age	− 0.143; 0.003; [− 0.522 to − 0.112]	–	− 0.113; 0.010; [0.054 to 0.393]	–	–	–	–	–	–
Education ^c^									
Senior middle school	0.105; 0.017; [1.330 to 13.648]	–	–	–	0.159; 0.004; [2.869 to 15.058]	0.111; 0.029; [0.917 to 16.375]	–	0.198; 0.002; [4.182 to 18.795]	0.136; 0.002; [22.304 to 96.643]
College	–	–	–	–	–	–	–	–	–
Occupation ^d^									
Blue collar	–	–	–	–	–	–	–	–	–
White collar	–	–	0.099; 0.021; [0.811 to 9.872]	–	–	–	–	0.146; 0.027; [0.801 to 12.817]	–
DAS28	− 0.738; < 0.001; [− 18.498 to − 14.381]	− 0.520; < 0.001; [− 18.823 to − 11.017]	− 0.822; < 0.001; [− 18.175 to − 14.642]	− 0.563; < 0.001; [− 10.541 to − 6.872]	− 0.647; < 0.001; [− 13.274 to − 9.475]	− 0.720; < 0.001; [− 18.154 to − 10.259]	− 0.459; < 0.001; [− 19.864 to − 15.047]	− 0.442; < 0.001; [− 9.857 to − 5.419]	− 0. 800; < 0.001; [− 120.414 to − 97.248]
*Model 3*									
*R*^*2*^	0.670	0.305	0.722	0.425	0.499	0.573	0.266	0.314	0. 728
Age	− 0.132; 0.004; [− 0.491 to − 0.094]	–	0.126; 0.003; [0.086 to 0.413]	–	–	–	–	–	–
Education ^c^									
Senior middle school	0.103; 0.016; [1.410 to 13.327]	–	–	0.112; 0.046; [0.111 to 10.981]	0.157; 0.003; [3.116–14.642]	0.110; 0.023; [1.196 to 15.889]	–	0.182; 0.003; [3.466 to 16.715]	0.135; 0.001; [25.888 to 91.587]
College	–	–	–	–	–	–	–	–	–
Occupation ^d^									
Blue collar	–	–	–	–	–	–	–	–	–
White collar	–	–	0.086; 0.038; [0.267 to − 9.002]	–	–	–	–	–	–
DAS28	− 0.630; < 0.001; [− 16.399 to − 11.670]	− 0.386; < 0.001; [− 14.810 to − 6.631]	− 0.714; < 0.001; [− 16.227 to − 12.289]	− 0.347; < 0.001; [− 7.447 to − 3.287]	− 0.469; < 0.001; [− 10.447 to − 6.036]	− 0.563; < 0.001; [− 16.446 to − 10.843]	− 0.291; < 0.001 [− 13.914 to − 4.597]	− 0.330; < 0.001; [− 8.228 to − 3.157]	− 0.600; < 0.001; [− 94.286 to − 69.142]
Overall BIPQ	− 0.193; < 0.001; [− 0.785 to − 0.241]	− 0.230; 0.002; [− 1.250 to − 0.273]	− 0.215; < 0.001; [− 0.739 to − 0.287]	− 0.379; < 0.001; [− 0.948 to − 0.451]	− 0.307; < 0.001; [− 0.908 to − 0.381]	− 0.270; < 0.001; [− 1.118 to − 0.446]	− 0.290; < 0.001; [− 1.722 to − 0.597]	− 0.272; < 0.001; [− 0.864 to − 0.259]	− 0.343; < 0.001; [− 7.080 to − 4.077]
*Model 4*									
*R*^*2*^	0.673	0.298	0.718	0.434	0.518	0.578	0.269	0.378	0. 740
Age	− 0.145; 0.002; [− 0.541 to − 0.147]	–	0.129; 0.003; [0.090 to 0.421]	–	–	–	–	–	–
Education ^c^									
Senior middle school	0.105; 0.014; [1.643 to 13.420]	–	–	0.113; 0.042; [0.218 to 11.024]	0.158; 0.002; [3.222 to 14.594]	0.111; 0.021; [1.312 to 15.947]	–	0.168; 0.004; [2.954 to 15.707]	0.139; < 0.001; [28.238 to 93.028]
College	–	–	–	–	–	–	–	–	–
Occupation ^d^									
Blue collar	–	–	–	–	–	–	–	–	–
White collar	–	–	0.091; 0.030; [0.480 to 9.293]	–	–	–	–	–	–
DAS28	− 0.699; < 0.001; [− 16.748 to − 12.129]	− 0.415; < 0.001; [− 15.455 to − 7.571]	− 0.742; < 0.001; [− 16.743 to − 12.895]	− 0.397; < 0.001; [− 8.103 to − 4.158]	− 0.460; < 0.001; [− 10.256 to − 5.935]	− 0.589; < 0.001; [− 16.971 to − 11.627]	− 0.341; < 0.001; [− 14.983 to − 6.082]	− 0.274; < 0.001; [− 7.086 to − 2.415]	− 0.610; < 0.001; [− 95.423 to − 70.438]
BIPQ–1 Consequences	–	–	–	–	− 0.135; 0.032; [− 1.989 to − 0.087]	–	–	–	–
BIPQ–3 Personal control	0.169; < 0.001; [0.780 to 2.781]	–	0.086; 0.038; [0.052 to 1.746]	0.225; < 0.001; [0.910 to 2.751]	0.134; 0.014; [0.263 to 2.214]	0.138; 0.006; [0.515 to 3.008]	–	–	0.119; 0.004; [2.857 to 14.194]
BIPQ–4 Treatment control	–	–	–	–	–	–	0.139; 0.027; [0.273 to 4.644]	–	0.084; 0.029; [0.640 to 12.391]
BIPQ–5 Identity	–	− 0.198; 0.007; [− 4.557 to − 0.751]	− 0.146; 0.002; [− 2.308 to − 0.508]	–	–	–	–	–	− 0.105; 0.034; [− 13.159 to − 0.430]
BIPQ–7 Coherence	–	–	–	–	–	–	–	0.127; 0.033; [0.079 to 2.134]	–
BIPQ–8 Emotional response	–	–	–	− 0.232; < 0.001; [− 2.488 to − 0.717]	− 0.180; 0.006; [− 2.387 to − 0.405]	− 0.193; 0.001; [− 3.276 to − 0.877]	− 0.224; 0.002; [− 5.061 to − 1.106]	− 0.390; < 0.001; [− 4.010 to − 1.940]	− 0.207; < 0.001; [− 18.334 to − 6.811]

SF-36 abbreviations: BP, bodily pain; GH, general health; MH, mental health; PF, physical function; RE, role limitations due to emotional problems; RP, role limitations related to physical problems; SF, social functioning

^a^The SF-36 dimensions reflecting HRQoL were treated as dependent variables

The selection of independent variables for multiple regression analyses was based on these analyses (Tables 2 and 3). Model 1, demographic variables (gender, age, education, type of residence, family income, employment)

Model 2, Model 1 + clinical characteristic variables (disease duration, DAS28); Model 3, Model 2 + overall BIPQ; Model 4, Model 2 + the 8 BIPQ domains without the overall BIPQ

^b^Reported as: *β*; *P*, 95% CI, unless indicated otherwise

Dummy Variables: ^c^ Reference as junior middle school; ^d^ Reference as unemployed

Four models were analyzed, with different variables. Model 1 included demographic variables (gender, age, education, type of residence, family income, and employment). Age, education, and occupation could account for 17.1% of the variance of the total SF-36 score (Model 1, R^2^ = 0.171, F = 12.855, *P* < 0.001). Model 2 included the demographic variables of Model 1, and in addition the clinical characteristics disease duration and DAS28. DAS28 could account for 47.9% of variance of the total SF-36, in addition to the demographic variables (Model 2, R^2^ = 0.650, F = 174.913, *P* < 0.001). Model 3 included all the variables of Model 2 (demographic variables plus disease duration and DAS28) and the overall BIPQ. (Model 3, R^2^ = 0.728, F = 167.215, *P* < 0.001). Model 4 incorporated Model 2 and the 8 BIPQ domains, without the overall BIPQ. (Model 4, R^2^ = 0.740, F = 87.37, *P* < 0.001).

In addition, the BIPQ domains personal control, treatment control, identity, and emotional response were associated with HRQoL when the demographic factors and disease characteristics were controlled. The BIPQ domains explained 9.0% of the variance of the total SF-36. Identity and emotional response were negatively associated with HRQoL, however personal control and treatment control were positively associated with HRQoL. Furthermore, consequences were associated with vitality, and coherence with mental health (Model 4, *P* < 0.05). The total BIPQ had a significant negative association with each component of the SF-36, and also the total SF-36 (Model 3).

## Discussion

This study investigated the illness perception and HRQoL of patients with RA in China, and associations between illness perception and each domain of HRQoL. It was found that the total BIPQ had a significant negative association with SF-36: as scores of illness perception increased, the quality of life worsened. Thus, illness perceptions were identified as likely targets for strategies to improve the quality of life of patients with RA.

This is the first report of the illness perceptions of patients with RA in China. The overall BIPQ score was 49.09 ± 11.06, which was higher than reported by a study from Greece (40.08 ± 1.06) [18]. This showed that the patients with RA in the present cohort had a more negative view of the disease than did patients in Greece. In western countries, the domain with the highest BIPQ score was timeline, and the lowest was identity [16–18]. The present study found that the highest BIPQ score was for illness concern and timeline. Our results showed that the timeline score was higher, in accord with previous studies on RA [17, 18]. Although the survey populations differed, these study comparisons show that patients with RA in China and in western countries generally recognize that RA is a chronic disease with a long course.

The present study also found that patients older than 45 years had significantly lower scores for physical function, vitality, and social functioning compared with younger patients. Patients with a disease duration more than 5 years, and more serious disease activity, had the worst HRQoL. Patients with higher education and engaged in white collar occupations had better HRQoL compared with patients with less education or poorer employment. A literature review reported that increased age was associated with reduced physical function and physical component summary scores for HRQoL [11]. The present analysis also showed that age was negatively associated with physical function, bodily pain, general health perception, vitality, and social functioning.

In addition, the multivariate analyses showed that disease activity was negatively associated with each of the SF-36 components. Education and DAS28 were associated with total HRQoL (Model 2, R^2^ = 0.650, F = 174.913, *P* < 0.001). It must be noted that clinical characteristics accounted for 47.9% of the variance in total HRQoL, in addition to the demographic variables (Table 4). These findings revealed that disease activity had a dramatic effect on the HRQoL of patients with RA. The data in the present study support that increased disease activity is associated with reduced HRQoL in RA[36, 37]. RA has also been noted to affect the HRQoL of patients, by the clinical manifestations of the disease, and by socioeconomic, personal, and environmental factors [4].

Berner et al. [27] reported that illness perception accounted for 51% and 45% of variance in physical and mental HRQoL, respectively. However, we found that total BIPQ was negatively associated with each of the SF-36 components (Model 3). In addition, the domains of the BIPQ explained 9.0% of the variance in total SF-36, besides clinical characteristics and demographic variables (Model 4, R^2^ = 0.740, F = 87.37, *P* < 0.001). Although this association is not strong, it obviously has importance. The reason for such discrepancy may be the different study populations and recruitment methods. It has also been reported that sleep impairment is a common clinical condition in patients with RA [38, 39], and has been confirmed to affect quality of life [40], the majority of Chinese patients with RA suffer from poor sleep and impaired quality of life [41]. Fatigue is highly prevalent in individuals with RA, and is perceived to have a significant detrimental effect on health status, and physical and social functioning [44], These are significant factors that affect the quality of life in patients with RA [12, 42], and further study is warranted.

Hyphantis et al. [17] reported that perceived consequences of the disease were independent correlate of physical HRQoL. The present study found that perceived consequences were associated specifically to vitality in HRQoL. The high scores indicated that patients with strongly held beliefs regarding the serious consequences of RA had worse vitality. Kotsis et al. [16] reported that anxiety, depression, and identity were associated with HRQoL in patients with RA, but not personal control or treatment control. However, the present multivariate analysis showed that personal control and treatment control were positively associated with HRQOL. Effective treatment and the ability of the patient toward self-management can control the disease, and improve the HRQoL. The difference in results may be related to differences in the survey population and the survey scales.

A review regarding illness perceptions concluded that negative emotions can affect the treatment of diseases, limit the physiological functions of patients, and seriously affect the prognosis of diseases. Illness perceptions, depression, anxiety, and quality of life were closely related [43]. Illness perception is associated with depression in patients with chronic illness [44]. Lu et al. [45] reported that components of illness perception were associated with negative emotions in depressed patients. During the COVID-19 pandemic, patients with RA were reported to experience severe anxiety and depression [46]. We propose that during this time patients with RA should be encouraged to enhance their illness perception and self-management. In so doing, their personal control of RA may increase, with fewer negative emotions, and HRQoL improve despite the pandemic. This deserves further study.

Ho et al. [44] reported that up to 52% of patients with RA experienced symptoms of anxiety and/or depression [44]. Liu et al. [47] found that the IL-17 level positively correlated with the severity of anxiety in patients with RA. A meta-analysis showed that patients with RA with depression tended to have lower quality of life than patients without depression [48]. Depressed patients with RA have more pain [49], high disease activity [50], and reduced HRQoL [2]. The present study also found that emotional response in patients with RA was negatively associated with HRQoL (physical function, general health perception, vitality, social functioning, role limitations due to emotional problems, and mental health). In addition, identity was negatively associated with HRQoL (role limitations related to physical problems, bodily pain). Our finding emphasizes that patients with RA who experience serious negative emotions caused by their disease usually have a heavier mental burden and worse quality of life.

This study specifically showed that illness perceptions influenced different dimensions of HRQoL in patients with RA. Patients with the worst illness perceptions had the worst HRQoL. Previous research in RA showed that illness perceptions had significant implications for adaptation to illness and notably affected medical disease status, even more so than depression, physical function, or pain [51]. Berner et al. [27] highlights the importance of patients’ beliefs about their illness and symptoms in relation to HRQoL. Identification of patients’ perception of RA may be a way to influence quality of life for the better. Health interventions based on understanding and modifying perceptions of illness proved useful in facilitating patient’s HRQoL [51, 52]. Illness perceptions can change over time, and these changes affect patients’ outcomes [53]. Patients with RA may benefit from illness perception modification. Future evidence-based interventions that focus on illness perception are required to enhance the HRQoL of patients with RA [54, 55].

## Limitations

There are some limitations to this study. The cases were from a single hospital and the sample size was relatively small. Longitudinal studies are needed before further conclusions can be drawn. Future studies should add variables such as sleep disturbance, depression, anxiety, and fatigue, and explore how these factors and illness perceptions can affect HRQoL.

## Conclusions

Illness perceptions were associated with the HRQoL in this study of a patient population with RA in China. Patients with the worst illness perceptions had the worst HRQoL. Illness perceptions are important potential targets to improve the quality of life of patients with RA. Illness perceptions may therefore be a useful basis for future quality of life interventions.

## Data Availability

The data used are under license for the current study. They are not publicly available.
